# Reply to ‘Thomson scattering in inhomogeneous plasmas: The Role of the Fluctuation-Dissipation Theorem’

**DOI:** 10.1038/s41598-018-25322-x

**Published:** 2018-05-21

**Authors:** P. M. Kozlowski, D. O. Gericke, S. P. Regan, G. Gregori

**Affiliations:** 1 Department of Physics, West Virginia University, Morgantown, WV 26506 USA; 2 Department of Physics, University of Oxford, Parks Road, Oxford, OX1 3PU United Kingdom; 3 Centre for Fusion, Space and Astrophysics, Department of Physics, University of Warwick, Coventry, CV4 7AL United Kingdom; 4 Laboratory for Laser Energetics, University of Rochester, 250 East River Road, Rochester, NY 14623 United States

## Abstract

In a comment on our article “Theory of Thomson scattering in inhomogeneous media”, V. V. Belyi asserts that there is an inconsistency in our method of applying gradient effects via the dielectric superposition principle, in violation of the fluctuation-dissipation theorem; and that his Klimontovich-Langevin formulation would be more appropriate to our application. While we agree that a generalization, along the lines of Belyi’s work, would be required for strongly coupled systems, for the weakly coupled systems which we considered, these corrections are not necessary and our approach is still appropriate.

Replying to: V. Belyi, *Sci. Rep*. 8 (2018); 10.1038/s41598-018-25319-6.

## Introduction

We thank Belyi for his comment on our manuscript. First, we would like to note that both our work and Belyi’s work find that strong modifications of the equilibrium structure factors are possible in inhomogeneous and relaxing media^1,2^. We agree that Belyi’s treatment is more general than ours, and indeed gradient corrections in both the densities and the fields must be included, in general. Such a treatment is particularly required for strongly coupled plasmas. However, it was never our intention to describe systems with strong interactions in the electron component. As we explicitly stated, Eq. (2) of our paper is derived in the limit of the random phase approximation (RPA) applicable for weakly interacting electrons, as they occur in many experiments^1^. This treatment is equivalent to the dielectric superposition principle, as was shown by Ichimaru^3^. In the RPA limit, Eq. (2) of our paper is the correct connection between the dielectric response and the dynamic electron structure factor. In the same limit, gradient corrections only apply to the screening dielectric function and not to the ideal structure factor - which is clearly unaffected by gradients. This behaviour is related to the fact that gradients generate nonlocal effects in the screening electric fields (via the nonlocal Poisson equation), whereas there is no such inter-particle field to carry nonlocal effects in the ideal gas^3^. For that reason, the gradient expansion is only acting on the dielectric function and not on the ideal dynamic structure factor. Of course, the summation of spatial domains with different conditions must still be done. Therefore, Eqs (2) and (4) are consistent in describing the effects of gradient corrections to the dynamic structure factor for weakly coupled plasmas, where the RPA is valid, with small perturbations.

In contrast to the considered case of weakly coupled plasmas, all contributions to the total structure factor should be subject to the gradient expansion for systems with moderate or strong interactions. In this case, the approximation of Eq. (2) becomes invalid and one needs to apply a more general treatment, e.g., as presented by Belyi^2^. Of course, these gradient effects, and therefore the differences between our theories, are only important for scattering parameters $\alpha ={k}_{D}/k > 1$, and both theories agree in the limit α <<1, where the scattering is dominated by single particle scattering and the additional correction terms due to the plasma gradients vanish.

In conclusion, we thank Belyi for pointing out a valuable extension to our work, that goes beyond the RPA limit and can describe a larger class of situations. We hope that both of our results will stimulate further studies on this topic.
